# Selective Laser Sintering of a Chitosan/MOF Composite: 3D-Printed Adsorbent for Arsenic

**DOI:** 10.1021/acsomega.5c06075

**Published:** 2025-11-11

**Authors:** Jessy Joseph, Ari Väisänen, Manu Lahtinen

**Affiliations:** Department of Chemistry, 4168 University of Jyväskylä, P.O. Box 35, FI-40014 Jyväskylä, Finland

## Abstract

Metal–organic framework/biopolymer composites have shown significant potential for removing pollutants such as arsenic from aquatic environments. However, due to the poor mechanical properties and challenges in incorporating these materials into existing water treatment systems, their use in real-world applications is limited. In this study, we developed mechanically strong porous filters for adsorbing arsenic (As­(III) and As­(V)) by utilizing the selective laser sintering (SLS) 3D printing technique. In the printed filter disks, polystyrene is used as the polymeric supporting matrix, and a bimetallic Mn-doped MIL-100­(Fe)-based MOF/chitosan composite CS-MIL-100­(Fe, Mn) (hereafter: CS/MOF) as the adsorptive filler, with 5, 10, and 15 wt % filler concentrations. With powder X-ray diffraction (PXRD), it was confirmed that the crystal phase of the CS/MOF is preserved in the printed filters. Scanning electron microscopy (SEM) revealed the hierarchical porous structure of the composite consisting of the meso- and macroporous channel networks formed by the partially sintered polystyrene particles, along with the micro- and mesopores of the CS/MOF adhered to the polymer surface. The filters efficiently removed As­(III) and As­(V), and the highest adsorption rate of 99% was achieved at the filler concentration of 15 wt %. The developed filter maintained its optimal adsorption efficiency toward arsenic species, even in the presence of multiple competitive anions such as sulfate, phosphate, nitrate, and carbonate. The filter also exhibited the potential for reducing the As­(III) and As­(V) concentrations from 2.5 mg/L to lower than 10 μg/L from tap water, which was modified to simulate a typical inorganic composition of arsenic-polluted groundwater.

## Graphical abstract

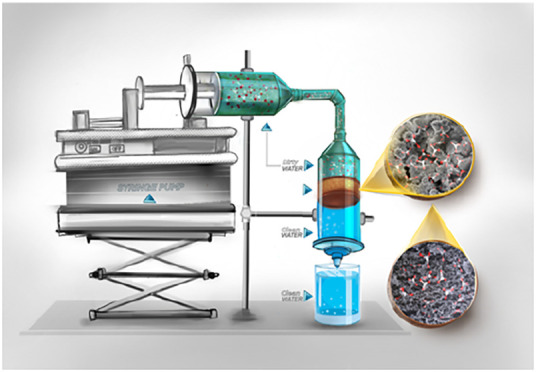

## 1 Introduction

Arsenic is one of the most dangerous naturally occurring contaminants, with severe effects on the world′s population. In natural water, arsenic is primarily present in two oxidation states, +3 and +5, and its speciation is highly dependent on the pH of the water. Among the different forms of arsenic, the inorganic forms pose the greatest challenge, specifically the As­(III), which pose greater difficulties to remove due to their nonionic nature and higher mobility.^1^ Arsenic is classified as a Group 1 carcinogen. Considering the health risks associated with arsenic, the World Health Organization (WHO) has recommended a guideline value of 10 μg/L for arsenic in drinking water.^2,3^ Various techniques have been used to remove arsenic, including coagulation and flocculation, precipitation with or without oxidation, ion exchange, membrane filtration, and electrodialysis.^4,5^ These methods often have significant drawbacks, which include the generation of secondary hazardous arsenic-containing sludge. Adsorption techniques have emerged as promising alternatives to the above-mentioned methods. Commonly, adsorbents, such as zeolites, carbon black, or various metal oxides, e.g., iron or aluminum oxides, are used for the adsorption of arsenic.^6,7^ However, the efficiency of these materials is generally limited, particularly in the adsorption selectivity of arsenite (As­(III)).

Metal–organic frameworks (MOFs), porous coordination polymers formed by the coordination bond between metal ions/clusters and organic ligands, have shown great potential for arsenic removal. Compared to conventional adsorbents, tunable porosity and enormous surface area make MOFs extremely effective as adsorbents. In addition, MOFs offer other exceptional characteristics, such as preselective or postsynthetic functionalization of the organic ligand and chemical tuning of the metal nodes to enhance the adsorption capacity and affinity toward guest molecules and ions.^8–11^ The superior arsenic adsorption capacity of metal–organic gel Fe-BTC, synthesized through the coordination of iron and 1,3,5-benzene tricarboxylic acid, compared to the commercial and nano iron oxide, as reported by Zhu et al., has initiated further investigation on the MOF’s potential for arsenic adsorption.^12^ Later, Cai et al. reported well-crystallized MIL-100­(Fe) MOF built from the same precursors, and the adsorbent demonstrated an adsorption capacity of 110 mg/g for As­(V).^13^ Zirconium-based MOF UiO-66 was also reported to be efficient for arsenic adsorption with a high 303 mg/g adsorption capacity for As­(V). Bimetallic MOFs are also gaining much attention in arsenic adsorption.^14^ Studies have reported that incorporating secondary metal centers is beneficial for arsenic adsorption. By incorporating La into Zr metal centers present in MOF-808, the adsorption efficiency for As­(V) increased from 110 to 217 mg/g of adsorbent.^15^ Similarly, Yin et al. reported that the Fe/Al bimetallic MOF Fe/Al-BDC-NH_2_ performed better than its monometallic counterparts.^16^ Despite the fascinating properties of various MOF materials, their utilization in water treatment still has major challenges due to their powder form. The powdered form of MOF causes loss of the active material, clogging of pipes, and time-consuming secondary separation processes to recover the used MOF from the aqueous medium.^17–20^ Therefore, developing immobilized MOF materials is crucial for practical and scalable applications. One potential solution for immobilization is MOF-biopolymer composites.

Chitosan, derived from chitin, shows significant potential as a polymeric support for a MOF.^21^ Chitosan also demonstrates efficiency in arsenic adsorption, especially in acidic and near-neutral pH.^22^ Chitosan composites have been reported to be efficient adsorbents for arsenic. Chitosan loaded with MgAl layered double hydroxide is reported to be an efficient adsorbent for As­(V).^23^ Similarly, chitosan/Fe_3_O_4_ nanocomposite prepared from chitosan and industrial waste red mud has shown promising performance in As­(III) removal.^24^ MOF/chitosan composites have been reported as highly efficient adsorbents. Wei et al. prepared an activated UiO-66/chitosan composite for As­(III) adsorption.^25^ A quaternary ammonium-modified ZIF-8/chitosan nanocomposite exhibited 54.2 mg/g of As­(V) adsorption capacity.^26^ We have reported a highly efficient and selective MOF/chitosan composite for arsenic adsorption that was formed by *in situ* synthesis of MIL-100­(Fe) MOF onto chitosan matrix.^27^ Very recently, we reported *in situ* synthesis of a bimetallic MOF/chitosan composite by doping Cu, Co, Ni, and Mn into MIL-100­(Fe) during the synthesis. Among these, the Mn-doped MIL-100­(Fe, Mn)/chitosan composite exhibited 100% improved adsorption kinetics for both arsenic species and was proven to be more efficient in arsenite adsorption by improving the adsorption capacity by 87% compared to the monometallic counterpart.^28^ MOF/chitosan composites are often prepared as aerogel beads and sponges. Such structural motifs often demonstrate enhanced adsorption capabilities due to their hierarchical porous structure, which improves the availability of active sites through their mesoporous structure.^29–32^ However, these materials typically suffer from low mechanical stability and fragility. Therefore, it is crucial to develop materials and methods where fragile MOF composites can be employed for arsenic adsorption without compromising their hierarchical porosity.^33^

Selective laser sintering (SLS) 3D printing offers a promising alternative for developing sophisticated composite systems that are rigid and mechanically strong for real-world applications. In SLS 3D printing, printing material is sintered layer by layer using the heat from a laser to build the desired objects.^34^ In this technique, metals and polymers can be used as the printing material, and adsorbent materials, e.g., metal oxide, ion exchange materials, and other functional fillers, can be incorporated by mixing them with the printing material.^35,36^ SLS 3D-printed materials have been studied for various water treatment applications such as metal recovery, trace element analysis, and pollutant removal.^37–40^ Ibebunjo et al. have reported the fabrication of Fe–Ni bimetallic adsorbent using SLS 3D printing for arsenic adsorption, exhibiting 0.95 and 0.921 mg/g of adsorption capacity for As­(III) and As (V), respectively.^41^

This additive manufacturing technique has proven to be effective in combining MOFs into polymer matrices without significantly compromising their intrinsic porosity. The printing parameters are optimized carefully to allow partial sintering of the polymer particles, thus generating a partially fused solid with macroscopic pores while retaining the structural properties of MOFs.^42^ Lahtinen et al. have reported the fabrication of HKUST-1/polyamide-12 disk using SLS for CO_2_ capture and elucidated that during the printing process, HKUST crystals are attached to the surface of the sintered particles. Thus, the HKUST-1 is available for adsorption even after printing.^43^ Similarly, a study was reported on the SLS-printed adsorbent for CO_2_ capture using *in situ* grown ZIF-8 on polyamide-12.^44^ Studies reported on the SLS-printed adsorbent using the MOF as a filler for water treatment are limited. To date, only one study has demonstrated the fabrication of a mixed matrix film using ZIF-67, NH_2_-MIL-101­(Al), MOF-801, HKUST, and ZIF-8 as a filler and studied its efficiency in removing methylene blue dye.^45^ To the best of our knowledge, no studies have reported the fabrication of MOF-based adsorbents using SLS 3D printing for arsenic adsorption. Additionally, no studies have reported the utilization of MOF/chitosan composites as fillers during the fabrication of adsorbents by using SLS 3D printing.

In this study, we developed SLS-printed disk-shaped adsorbents for removing arsenic from water. *In situ*, formed MOF/chitosan composite was used as the active filler material. The MOF employed here is a bimetallic MOF (MIL-100­(Fe, Mn)) formed by introducing dopant metal (Mn^2+^) into a crystal lattice of iron-based MIL-100­(Fe) MOF, which we had developed in our previously reported study.^28^ Polystyrene served as a polymeric support, enabling printing of an efficient disk-shaped filter for arsenic adsorption.

## 2 Experimental Section

### 2.1 Materials

Medium to high molecular weight chitosan (from shrimp shells, ≥75% deacetylated) was used having MW of 100,000–300,000 Da (g/mol), practical grade, and viscosity of 800–2000 cP (1% in 1% acetic acid at 25 °C), trimesic acid (H_3_BTC, 98%), 2-amino-2-(hydroxymethyl)-1,3-propanediol (Trizma base, ≥99.9%), and methanol, which all were obtained from Sigma-Aldrich. Iron­(III) nitrate nonahydrate (Fe­(NO_3_)_3_·9 H_2_O, ≥98%), hydrochloric acid (37%), and glacial acetic acid (100%) were procured from VWR AnalaR NORMAPUR ACS. Manganese­(II) nitrate tetrahydrate (≥98.5%) was purchased from Supleco and sodium hydroxide (98%) was from Fluka Chemical Corp. Arsenic trioxide (99.99%) and sodium arsenate diabasic heptahydrate (98%) obtained from Alfa Aesar and Millipore water were used in this study. The polystyrene (trade name Coathylene Sint PS) used in 3D printing was obtained from Axalta Polymer Powders Switzerland.

### 2.2 Synthesis of Chitosan/MOF Composites

The functional filler CS-MIL-100­(Fe, Mn) (hereafter represented as CS/MOF) was synthesized according to the reported method with a slight modification.^27,28^ The chitosan/metal solution was prepared by dissolving 9 mg/mL chitosan in 2% (v/v) acetic acid solution, followed by adding nitrate salts of corresponding metals to achieve final concentrations of 2.47 mmol for Fe^3+^ and 0.13 mmol for Mn^2+^ per gram of chitosan, respectively. The prepared chitosan-metal solution was introduced dropwise to 1 M NaOH solution, resulting in the immediate formation of the hydrogel beads. After the beads had undergone solidification in the NaOH bath for 2 h, they were washed with water until the eluent reached a neutral pH. The obtained hydrogel beads were subjected to methanol–water mixtures by gradually increasing the concentration of methanol (20:80, 50:50, 80:20 (v/v)) and finally twice in 100% methanol, each step lasting for 30 min for exchanging the synthesis and washing solvents. The obtained solvogel beads were reacted with 2.3 mmol of trimesic acid methanol solution in a Teflon-lined autoclave at 140 °C for 20 h, followed by cooling to room temperature. The formed CS/MOF composite beads were washed with methanol to remove unreacted and loosely bound reactants. Washed beads were gradually solvent exchanged to water and subjected to freeze-drying at 0.5 bar and −55 °C overnight.

### 2.3 3D Printing of Composite Filters

CS/MOF beads were used as the functional material in the 3D-printed filters. First, beads were hand-ground gently to a powder and sieved to limit the particle size to <125 μm, then were homogeneously mixed with polystyrene to obtain a powder mixture suitable for SLS printing. Filters with 5, 10, and 15 wt % of CS/MOF were printed using a Sharebot SnowWhite 2 SLS 3D printer. The shape of the filter was designed to be a cylindrical disk with 5 mm in height and 16.6 mm in diameter using FreeCAD. Each layer of the filter was sintered with a thickness of 100 μm at a rate of 45000 pulses per second (pps). The temperature of the printing chamber was maintained at 80 °C, and the printing temperature was fixed at 90 °C to prevent complete melting of the polystyrene (mp. 240 °C). The power of the laser varied with the concentration of the filler. Laser power was set to be 34%, 39%, and 44% of the maximum laser power (14 W) for 5, 10, and 15 wt %, respectively. Each filter disk was washed thoroughly by passing 5 mL of water through the filter at a flow rate of 90 mL/h to remove unsintered powder on the surface before being employed for the adsorption tests.

### 2.4 Characterization of Printed Filters

To investigate the effects of the printing process on the crystallinity of MIL-100­(Fe, Mn), filters were thoroughly examined by using powder X-ray diffraction (PXRD). The diffraction intensities between 2θ range of 3–40° were recorded using Panalytical X′Pert PRO MPD powder X-ray diffractometer with Cu Kα radiation (λ = 1.5418 Å) generated by a sealed X-ray tube (45 kV, 40 mA) and Ni β-filter. The program X′Pert HighScore Plus (v. 4.9) was used to analyze PXRD data. Fourier transform infrared spectrometer Nicolet iS50 (Thermo Fisher Scientific) in an ATR mode in the range 400–4000 cm^–1^ was used to characterize the functional groups in the filter material. The internal morphology and the dispersion of filler in the filters were imaged using scanning electron microscopy (SEM) (Raith e-LiNE). Energy-dispersive X-ray spectroscopy (EDX) analysis of the filters after arsenic species adsorption was performed with a Bruker Quantax400 connected to the Zeiss EVO-50XVP SEM instrument Zeiss EVO-50XVP.

### 2.5 Adsorption Experiments

As­(III) and As­(V) stock solutions were prepared from As_2_O_3_ and Na_2_HAsO_4_·7H_2_O, respectively. The preliminary adsorption experiments were done using a 2-filter setup, wherein two printed filter disks were mounted tightly on top of each other in the center of a 10 mL plastic syringe. The initial concentration of the arsenic solutions used in the tests was 5 mg/L. The solution was introduced into the filters at a flow rate of 50 mL/h, facilitated by a peristaltic syringe pump. Eluents were collected at 5 mL intervals, and the final arsenic concentrations were measured. The pHs of the As­(III) and As­(V) solutions were maintained at pH 9 and 7, respectively, if not stated otherwise, The selected pH values were based on our previously reported study in which the bimetallic and the monometallic counterparts of the filler used in the current work exhibited maximum adsorption of As­(III) and As­(V) at pH 9 and 7, respectively.^27,28^ Based on preliminary analyses, adsorption isotherm studies were conducted to determine the maximum adsorption capacity of the filter using a filter containing 15 wt % of CS/MOF with one filter disk in the syringe setup. 20 mL of arsenic solution at concentrations 10, 20, 40, 80, and 160 mg/L was pumped through the filters for adsorption isotherm studies at a flow rate of 10 mL/h.

The selectivity of the filter toward arsenic species in the presence of competitive anions was determined using a multianion test solution containing 10 mg/L of arsenic with equivalent concentrations of phosphate, sulfate, nitrate, and carbonate ions (one filter, flow rate 10 mL/h). The practical applicability of the filter was assessed using a simulated arsenic-contaminated solution prepared based on a reported study on the composition of groundwater in Bangladesh.^46^ The analyte solution was prepared by adopting the highest concentration of contaminants reported in the literature. The composition of the simulated water samples was: 2.5 mg/L of As­(III)/As­(V), 3 mg/L of sulfate, 0.22 mg/L of nitrate, 8.75 mg/L of phosphate, 21 mg/L of Ca^2+^, and 320 mg/L of bicarbonate, The solutions were prepared in tap water, and the pH of the solution was 6.7 (two filters, flow rate 10 mL/h). All adsorption studies were conducted in duplicate, and the results are reported as the average ± SD.

The arsenic concentrations were mainly measured using an inductively coupled plasma-optical emission spectrometer (ICP-OES), a PerkinElmer Avio 500. Arsenic concentrations below 100 μg/L were measured with an inductively coupled plasma-mass spectrometer (ICP-MS), a PerkinElmer NexION 350D.

The adsorption % and adsorption capacities (*q*_e_) were calculated based on eqs 1 and 2

1 $$adsorption\;\left(\%\right)={\frac{C_{o}-C_{e}}{C_{o}}}^{\;}\times 100$$

2 $$q_{e}={\frac{C_{o}-C_{e}}{W}}^{\;}\times V$$

The initial and equilibrium concentrations of the analyte are denoted as *C*_o_ and *C*_e_ (mg/L). *V* and *W* are the volumes of the analyte in liters and the dry mass of the adsorbent in grams, respectively.

## 3 Results and Discussion

The printed cylindrical filter disks with 0, 5, 10, and 15 wt % of CS/MOF (denoted as PS, PS-CS/MOF(5), PS-CS/MOF(10), and PS-CS/MOF(15), respectively) are shown in Figure 1. In this study, the maximum concentration of filler used is 15 wt %, as higher filler concentrations could not be used due to the inability to achieve a technically sufficient quality 3D prints. The effects of the printing process on the structural properties of CS/MOF were investigated by using PXRD. In Figure 2, PXRD patterns of PS (on top) and CS/MOF phases (at bottom) measured from powdered beads are shown. The MOF phase of the composite at the bottom represents the characteristic diffraction pattern of isostructural MIL-100­(Fe) crystal structure (CSD entry: CIGXIA) with the strongest peak intensities on 2θs of around 4° and 10–11°. Whereas the amorphous chitosan component demonstrates only a weak diffraction hump above baseline centered at 20.4° 2θ.^47,48^ Like amorphous chitosan, the PS is also amorphous, thereby showing only two very broad diffraction peaks centered at 9.4° and 19.2° 2θ in its PXRD pattern.^49^ The PXRD patterns of the filters exhibit characteristic peaks corresponding to both main components, as shown in Figure 2. In the case of the filter PS-CS/MOF(5), only the strongest diffraction peaks corresponding to the MIL-100­(Fe, Mn) crystal phase can be seen around 2θ 3.3° and 4°. With higher filler concentrations, e.g., 10 and 15 wt %, the diffraction peaks of the MOF phase were intensified and more distinctively seen on top of the broad diffraction humps originating from the polystyrene matrix. This confirmed that the adopted sintering condition for printing preserves the structural property, e.g., the crystallinity of the MOF phase across all of the printed filters.

**Figure 1:**
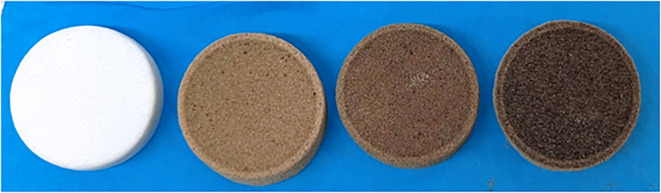
3D-printed filter disks: PS, PS-CS/MOF(5), PS-CS/MOF(10), and PS-CS/MOF(15).

**Figure 2:**
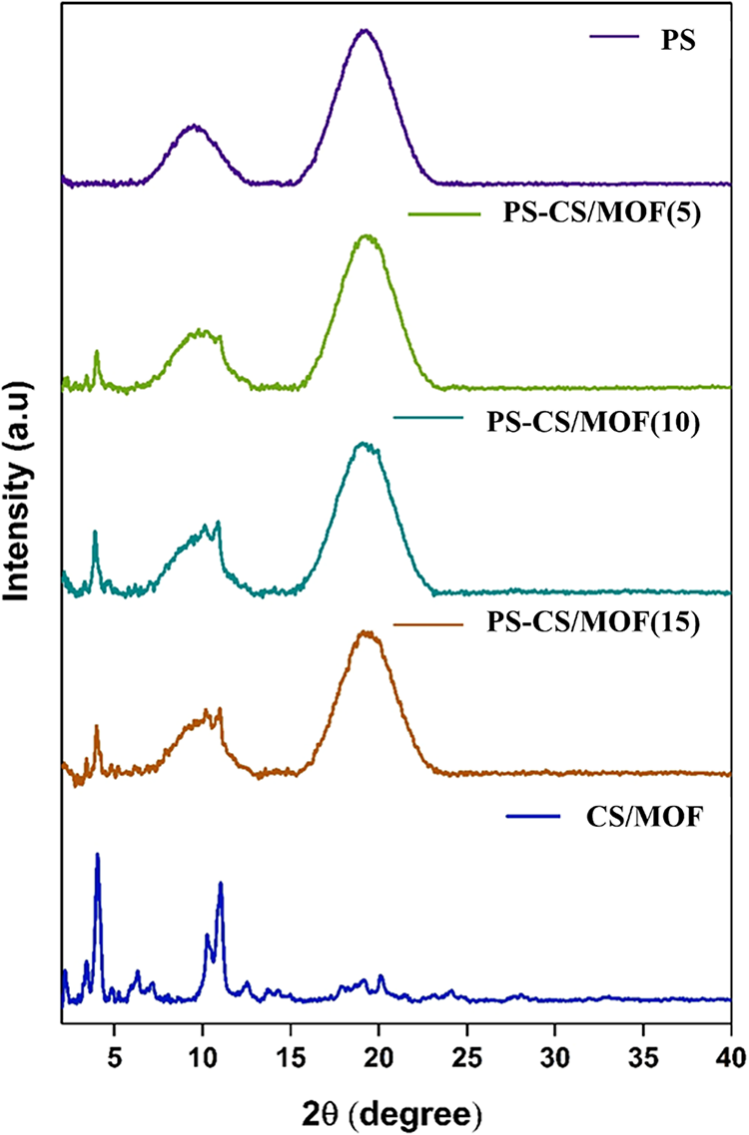
PXRD patterns of the 3D-printed filters with varying filler concentrations, PS, PS-CS/MOF(5), PS-CS/MOF(10), PS-CS/MOF(15), and CS/MOF (beads).

The vibrational modes of the composite filters were compared to those of PS and CS/MOF to understand the chemical integrity of the components after laser sintering (Figure 3). The prominent peaks of the printed filters originated from the polystyrene matrix. Nonetheless, the FTIR spectra of the filters with filler also exhibit characteristic features of the CS/MOF. A distinctive broad peak centered around 3350 cm^–1^ observed in the printed filters is due to the abundant OH groups in the chitosan. The emergence of a weak spectral band at 457 and 483 cm^–1^ in the spectrum of PS-CS/MOF(5), which becomes prominent at higher concentrations, represents the characteristic Fe–O vibration. This indicates that the integrity of the MIL-100­(Fe, Mn) framework present in the filler is preserved during the sintering process.^28^ Furthermore, the presence and stability of the CS/MOF component while printing is confirmed by the characteristic C=O vibration peaks at 1625 and 1570 cm^–1^, arising from the organic ligand (trimesic acid) of the MIL-100­(Fe, Mn) phase. The correlation observed between the intensity of the vibration peaks of the fillers and the weight ratio of the filler employed in each print confirms the proper integration of the fillers into 3D-printed filters.

**Figure 3:**
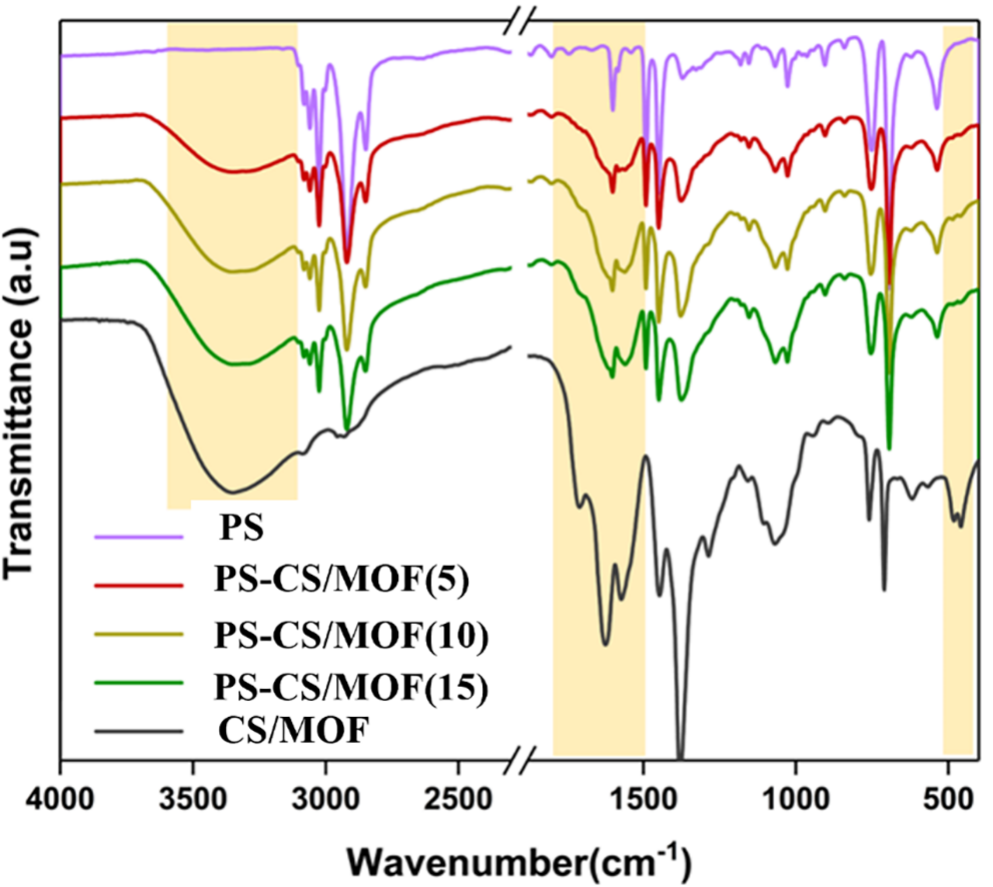
FTIR spectra of the printed filters PS, PS-CS/MOF(5), PS-CS/MOF(10), PS-CS/MOF(15), and CS/MOF (beads).

The cross-sectional SEM images of the CS/MOF show that the composite consists of a macroporous open channel structure (Figure 4). Closer examination reveals pore walls with embedded MOF crystals, confirming the presence of MIL-100­(Fe, Mn) within the composite.

**Figure 4:**
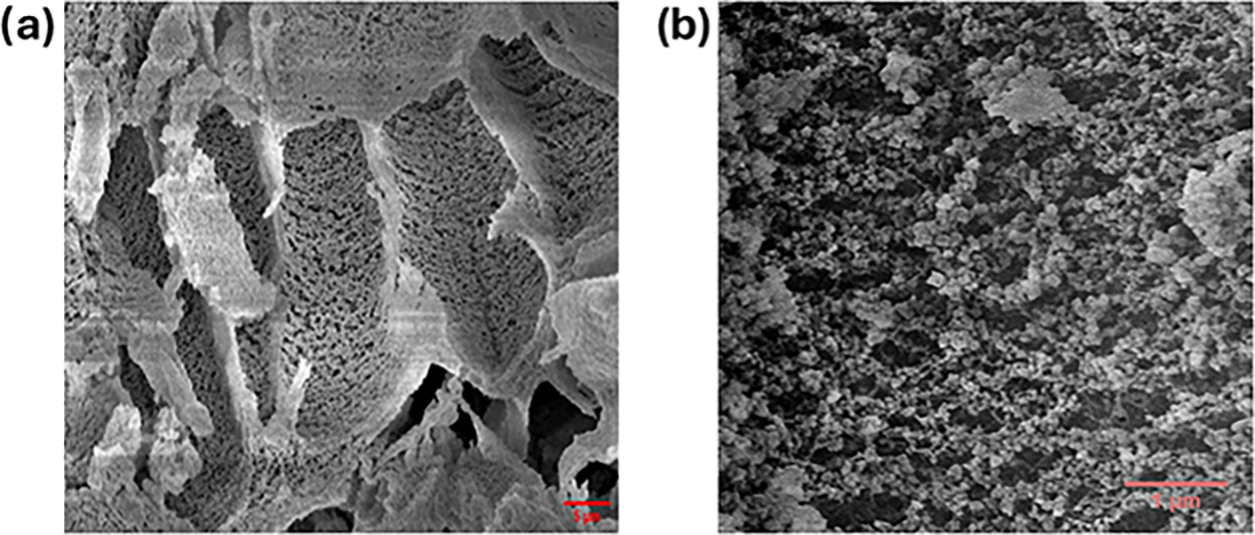
SEM images showing internal morphologies of the functional filler CS/MOF from scale bars (a) 5 and (b) 1 μm (left to right).

The internal morphology of the printed filter (Figure 5) demonstrates partially sintered polystyrene particles, and as a consequence, a highly porous structure with voids and flow channels has been generated. This confirms that the optimal parameter set was found for printing. Especially, the temperature seems to be proper since macroscopic pore structure is generated without extensive melting or significant merging of the polymer particles. The surface of the polymer particles in a pristine filter appears smooth. In contrast, the fillers are distinctly visible, as they are attached firmly to the surfaces of the polymer matrix. With the higher filler concentrations, better surface coverage was also achieved. This indicates that the functional fillers are not encapsulated by the partially fused polystyrene particles and thereby remain accessible to arsenic during adsorption.

**Figure 5:**
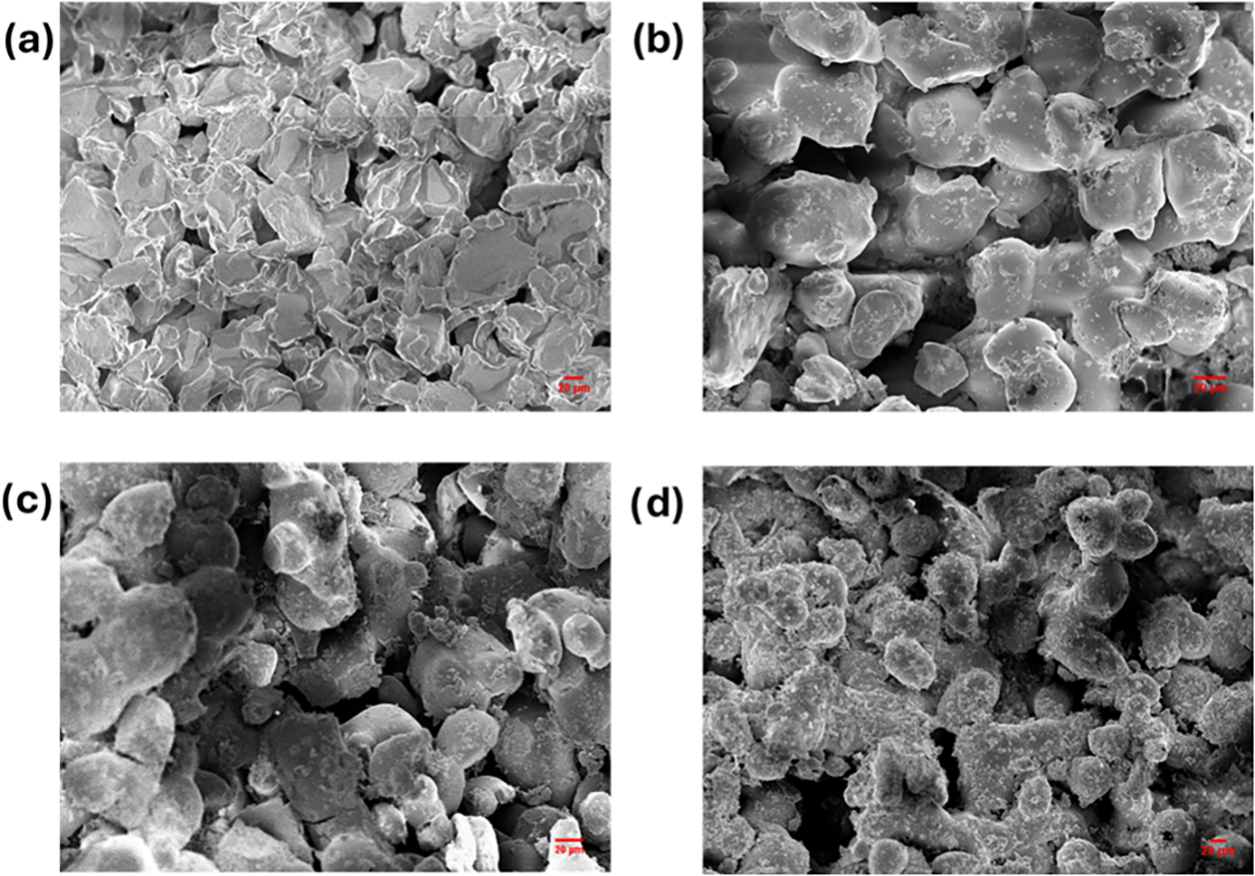
SEM images of the 3D-printed filters (scale 20 μm): (a) PS, (b) PS-CS/MOF(5), (c) PS-CS/MOF(10), and (d) PS-CS/MOF(15).

As can be seen from the SEM images, with an increase in filler concentration, the structure of the polystyrene powder becomes more spherical and highly fused. This may result from the higher laser power employed during the printing process along with the increase in the filler concentration. Nevertheless, the filter’s porosity is maintained, ensuring free liquid flow through it. This guarantees the formation of a fully functional product with a solvent-impermeable and chemically active interior across all three weight percentages. To understand the elemental composition of the 3D-printed filter, the EDX elemental composition analysis of the PS-CS/MOF(15) was conducted, and the results are reported in Figure S1. The EDX analysis revealed the presence of carbon, oxygen, nitrogen, manganese, and iron in percentage ratios of 60.3, 18.26, 19.67, 0.33, and 1.44, respectively.

Preliminary adsorption studies were conducted by using a two-stacked filter disk in a syringe, as shown in Figure 6.

**Figure 6:**
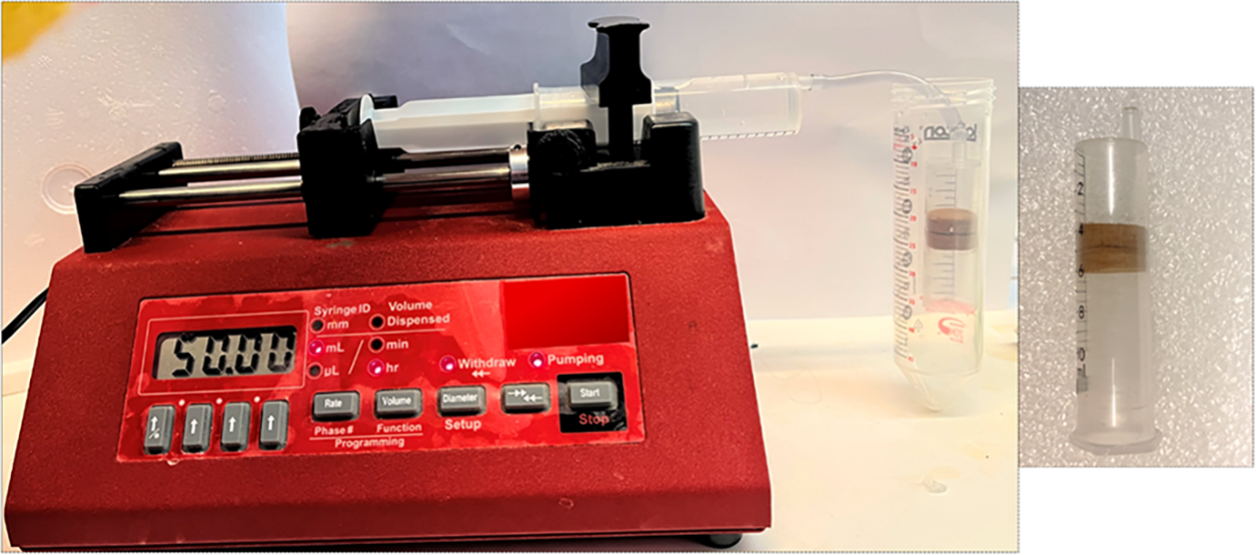
Experimental setup used for adsorption studies shows the syringe pump with arsenic stock solution connected to a two-stacked filter disk in the syringe.

The pristine PS filter exhibited zero adsorption capacity toward arsenic. Whereas the removal efficiency of arsenic increased rapidly with an increase in the filler concentration from 5 to 15 wt % (Figure 7). In detail, the filter with PS-CS/MOF(5) was effective in the 95% removal of As­(V) from the initial 10 mL influent volume. Whereas filters PS-CS/MOF(10) and PS-CS/MOF(15) removed 99% of As­(V) up to 25 and 65 mL of influent, respectively. PS-CS/MOF(15) was efficient enough to reduce the initial 5 mg/L concentration of As­(V) solution below the 10 μg/L level, up to 30 mL of solution filtered through the filter, thus falling below the maximum concentration of arsenic in drinking water recommended by WHO. To compare the adsorption efficiency of the three adsorbents, breakthrough volume defined as the eluted volume at which C_t_/C_0_ is 0.1, was determined and compared. It could be observed that the breakthrough volumes of the three tested filters are increasing on the order of 13, 40, and 95 mL for PS-CS/MOF(5), PS-CS/MOF(10), and PS-CS/MOF(15), respectively. This demonstrates that a 3-fold improvement in adsorption efficiency was observed by doubling the filler concentration. This indicates that the improvement in efficiency is a nonlinear function of filler concentration, and a relatively small increase in filler content would result in a disproportionately large improvement in the adsorption performance.

**Figure 7:**
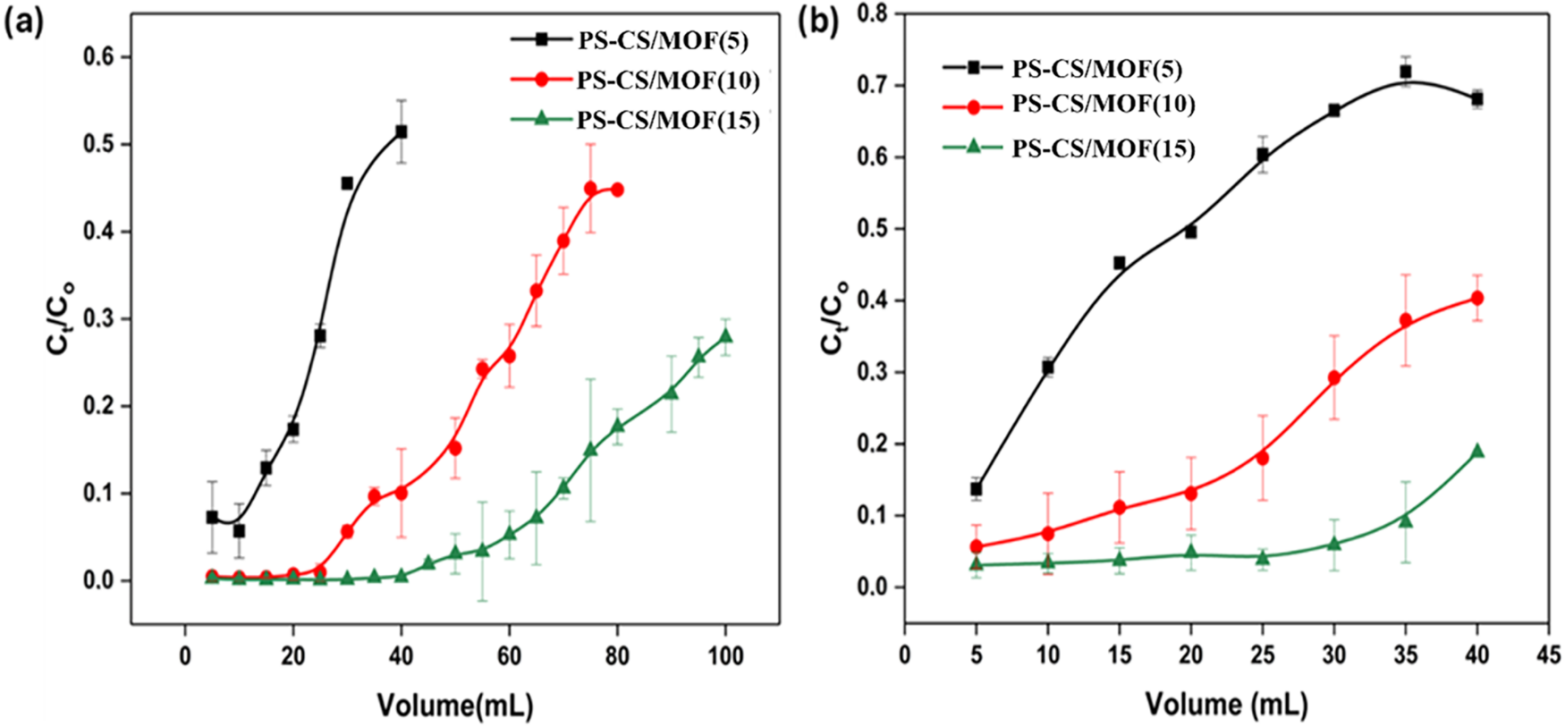
Adsorption performance of PS-CS/MOF(5), PS-CS/MOF(10), and PS-CS/MOF(15) filters in the removal of (a) As­(III) and (b) As­(V).

While inspecting the adsorption efficiency for As­(III), it can be noted that the filters, in general, perform slightly less effectively in comparison to their performance for As­(V). PS-CS/MOF(5) filter accomplished 90% adsorption of As­(III) from the 5 mL volume when the initial concentration of the solution was 5 mg/L. However, the adsorption efficiency reduces to 30% with a 40 mL influent volume. With an increase in filler concentration, a positive correlation in the adsorption efficiency was observed, as the PS-CS/MOF(10) exhibited 90% adsorption of As­(III) up to 10 mL volume. Adsorption declined gradually on higher volumes but remained close to 80% removal until 25 mL volume, eventually lowering to 60% after 40 mL of As­(III) solution was passed through the filter. PS-CS/MOF(15) maintained around 95% adsorption efficiency until 30 mL volume, then lowered to about 90% after 35 mL of solution was passed through the filter. In the case of As­(III), the breakthrough volumes for PS-CS/MOF(10) and PS-CS/MOF(15) were 13.5 and 35 mL, respectively. However, for the PS-CS/MOF(5) filter, the C_t_/C_0_ ratio reached 0.13 within the initial 5 mL flow volume.

To understand the effects of arsenic concentration on the absorptivity of the filters, the concentration range 1–160 mg/L was tested only with the best-performing filter PS-CS/MOF(15). For each concentration, 20 mL of As­(III) and As­(V) containing solutions was passed through a single filter disk setup inside the syringe at a flow rate of 10 mL/h. It was observed that the adsorption capacity of the filter toward both arsenic species increased with an increase in the initial arsenic concentration. This is assumed to be caused by more efficient access to the available active adsorption sites on the filter, as well as reduced mass transfer resistance due to the high driving force at higher arsenic concentrations. To further analyze the nature of the interaction between the filter and the arsenic species, the adsorption data obtained at different initial concentrations were studied using the Langmuir and the Freundlich adsorption models (eqs 3 and 4):

3 $$\frac{C_{e}}{q_{e}}=\frac{C_{e}}{q_{m}}+\frac{1}{K_{L}q_{m}}$$

4 $$q_{e}=K_{F}C_{e}^{1/n}$$

where *C*_e_ (mg/L) is the equilibrium concentration of arsenic species, *q*_m_ (mg/g) and *q*_e_ (mg/g) are the maximum adsorption capacity and the equilibrium adsorption capacity, respectively. *K*_L_ (L/mg) and *K*_F_ (mg/g­(L/mg)^1/*n*^) are the Langmuir and Freundlich constants, respectively.

The fitted isotherm models are shown in Figure 8, and the fitting parameters are given in Table 1. The Freundlich isotherm best describes the adsorption of arsenic species onto the filter by implying that the adsorption occurs via multilayer chemisorption on the heterogeneous active surfaces within the filter. This further supports the improved adsorption capacity at high initial As concentrations. The Freundlich constant *n* > 1 for both arsenic species indicates that heterogeneous adsorption is favorable across the entire concentration range on the filters. The Temkin isotherm model was also studied as it would address indirect adsorbate–adsorbent interactions during the adsorption process (eq S1 and Figure S3). The resulting analysis exhibits a poor fit compared with the Freundlich isotherm. In the Temkin isotherm, it is also assumed that the heat of adsorption of all molecules in the layer decreases linearly because of the increased surface coverage as the adsorbate occupies the heterogeneous adsorption sites. The constants in the Temkin isotherm B denote the Temkin constant related to the heat of adsorption, and A represents the equilibrium constant of the binding energy. While comparing the values of A, it could be observed that As­(V) has a stronger affinity toward the adsorption sites. However, the B, Temkin constant related to the adsorption heat of the adsorption value is larger for As­(III), indicating that the decrease in adsorption heat is slower for As­(III) compared to As­(V), showing stronger interaction with the adsorption site.^50^

**Figure 8:**
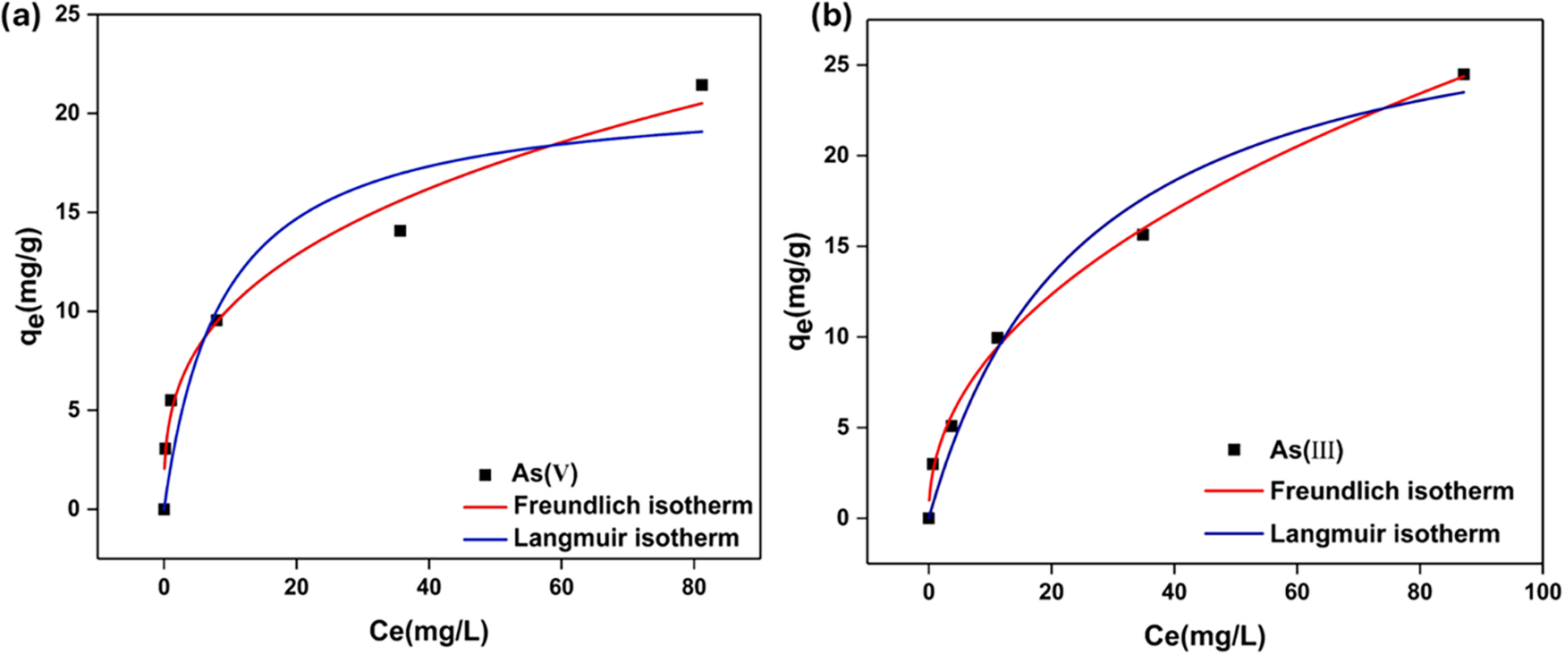
Effect of (a) As­(V) and (b) As­(III) concentrations on the adsorption performance of PS-CS/MOF(15).

**Table 1:** Fitted Langmuir and Freundlich Adsorption Isotherm Parameters of the PS-CS/MOF(15) for As­(III) and As­(V) Adsorption

	Langmuir isotherm			Freundlich isotherm		
	*q*_max_ (mg/g)	*K*_L_ (L/mg)	*R* ^2^	*K*_f_ (mg/g(L/mg)^1/*n*^)	*n*	*R* ^2^
As(III): relative to filler	30.2	0.0400	0.9721	3.09	2.16	0.9969
As(III): relative to filter	4.5	0.0400	0.9721	0.46	2.16	0.9969
As(V): relative to filler	21.1	0.1135	0.8994	4.74	3.00	0.9826
As(V): relative to filter	3.1	0.1135	0.8994	0.71	3.00	0.9826

Even though the Langmuir type of isotherm model exhibited a poor fit, especially for the adsorption of As­(V), the maximum adsorption capacities of PS-CS/MOF(15) were calculated using this model to find out the filter’s overall absorption capacity. For As­(III), the maximum adsorption capacity was determined to be 4.5 mg/g against the total filter weight. However, by normalizing it using the actual filler weight fraction, the capacity is 30.2 mg/g. For As­(V), the adsorption capacity is slightly lower compared to As­(III), as the maximum adsorption capacity was determined to be 3.1 and 21.1 mg/g in proportion to the total filter weight and the normalized filler weight, respectively. The previously determined negligible adsorption capacity for the pristine polystyrene indicates that it is solely in the role of support, and the adsorption capacities originate from the filler fractions. The contact area of the 3D-printed filter, calculated from the radius and the height of a disk, is 6.94 × 10^–4^ m^2^, and the total volume of As­(III) solution which could be treated per unit area of the filter is 648 L/m^2^, and for As­(V), it is 446 L/m^2^, at an initial arsenic concentration of 5 ppm.

The selectivity of the filter for arsenic determines its overall performance in removing arsenic species. Typically, common anions, such as sulfates, carbonates, nitrates, and phosphates, compete significantly with arsenic adsorption. The PO_4_^3–^ anion in particular can be harmful because it has a similar complexing ability, molecular structure, and chemical properties to arsenate, especially at neutral pH. Therefore, it is crucial to determine the potential interferences posed by these anions in the adsorption process. The interference of anions was studied by passing a multianion solution containing 10 mg/L of the target arsenic species (As­(III) at pH 9 or As­(V) at pH 7) with 10 mg/L of competitive anions such as sulfate, carbonate, phosphate, and nitrate through a one filter system at flow rate 10 mL/h. The results shown in Figure 9a depict that the developed filter exhibits no significant reduction in adsorption capacity, especially in the case of As­(III), for which the adsorption efficiency decreased by only 3%. In the case of As­(V), the adsorption % reduces by 7%. Notably, the filter maintained over 90% adsorption efficiency for both arsenic species, despite the presence of equimolar concentrations of competing anions. This demonstrates that the filter is efficient and highly selective toward arsenic, even in complex aqueous environments.

**Figure 9:**
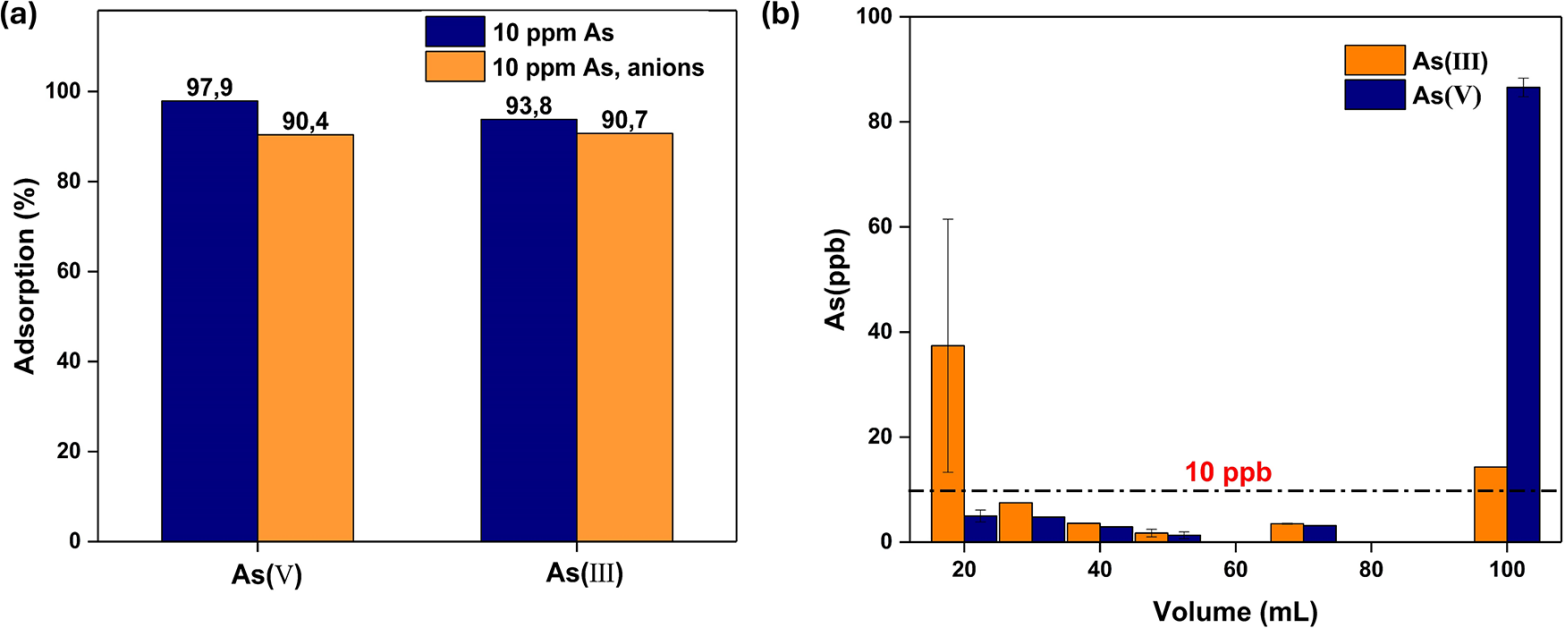
(a) Effect of competitive anions on the adsorption performance of PS-CS/MOF(15) and (b) filter performance under the mimicked groundwater conditions.

To further investigate the applicability of the developed filter in a real water system, experiments were conducted with modified tap water to simulate the inorganic composition of groundwater in Bangladesh (Figure 9b). In the case of As­(III), within the initial 20 mL of arsenic solution that passed the filter, the concentration was reduced from 2.5 mg/L to 37 μg/L. However, the concentration was further reduced to below the WHO′s recommended arsenic limit of 10 μg/L, which level was maintained up to 70 mL volume passed through the filter. Subsequently, as the eluted volume reached 100 mL, the remaining As concentration slightly increased to 14 μg/L. In the case of As­(V), the filter exhibited a similar performance. The As­(V) concentration was reduced below 10 μg/L within the initial volume of 20 mL and maintained a similar efficiency until 70 mL, but it increased to 86 μg/L as it reached 100 mL. Thus, it could be said that the filter efficiently removes arsenic below the WHO-recommended limit until a 70 mL volume was passed through the filter.

The SEM/EDX analysis of the filters (Figure 10a,b) after the adsorption of As­(III) and As­(V) confirmed the retention of CS/MOF on the PS surface, thus proving that during sintering the CS/MOF is firmly attached to the PS, facilitating the stability to resist the possible leaching throughout the adsorption process. EDX elemental mapping confirms the localized distribution of As­(III) and As­(V) adsorbed on the CS/MOF, confirming its availability for the arsenic species.

**Figure 10:**
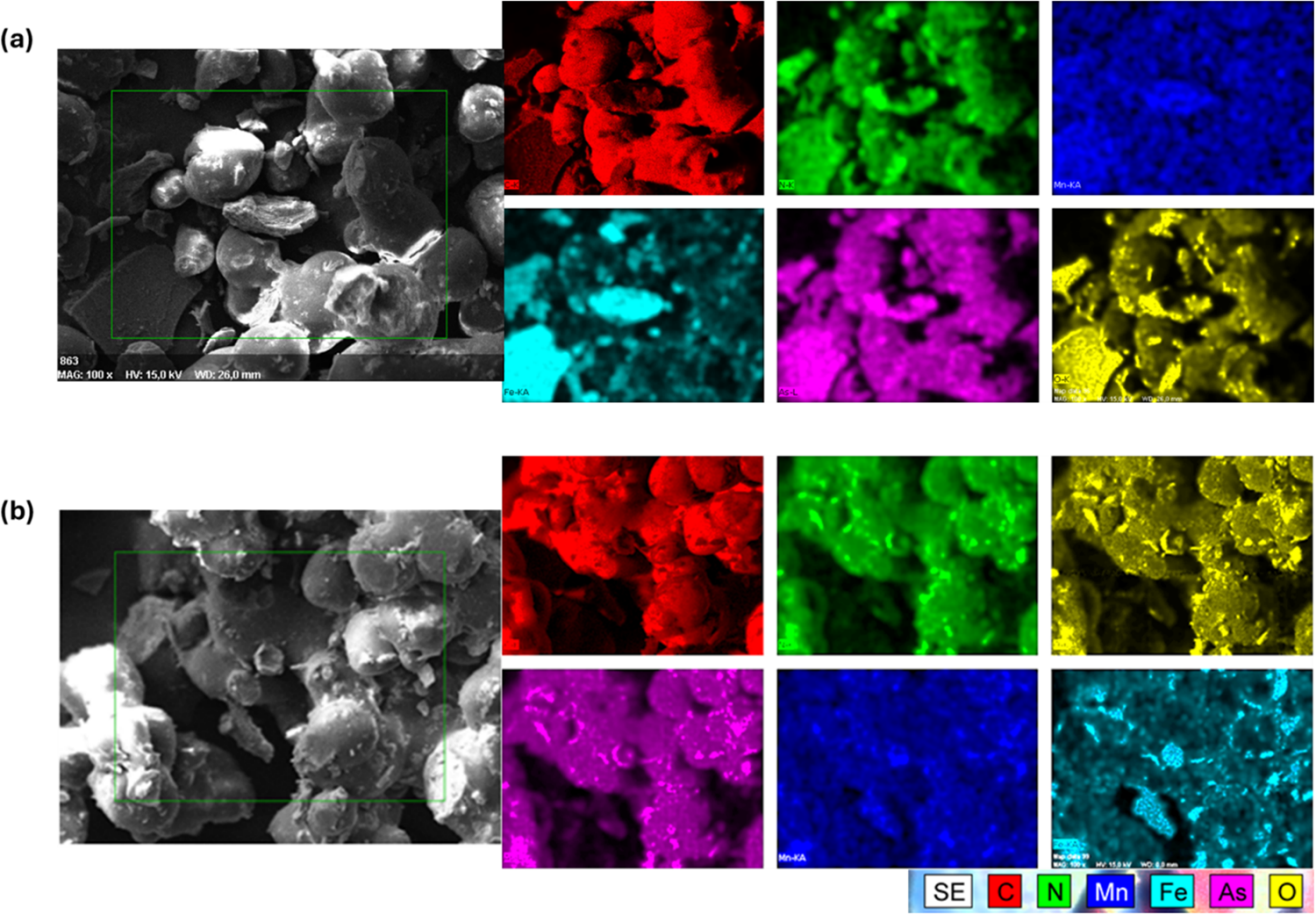
SEM/EDX image of the PS-CS/MOF(15) studied at 100× magnification after adsorption of (a) As­(III) and (b) As­(V).

## 4 Conclusions

This study introduces the fabrication of MOF composite filters through a powder-based SLS 3D printing technique for the efficient adsorption of arsenic. The printing powder mixtures were prepared by mixing CS/MOF at three different weight percentages: 5, 10, and 15 wt % to the polystyrene powder matrix. These filters are easily customizable and can be integrated into practical arsenic-purifying systems. The PXRD studies confirmed that the structural properties, e.g., the crystallinity of CS/MOF, were preserved during the laser sintering. The morphological analysis using SEM shows that SLS 3D printing is an effective method for developing adsorbents for pollutant adsorption. The internal porous structure generated as a result of the partial sintering of the polymer acts as a network of flow channels, facilitating an enhanced interaction between arsenic and the CS/MOF active sites that are firmly attached to the surface of the polymer. The arsenic adsorption tests conducted using the printed filters confirm the high performance of the developed filters and a positive correlation between CS/MOF wt % and arsenic adsorption efficiency. Among the studied filters, the filter PS-CS/MOF(15) exhibited a superior arsenic removal capacity. It achieved 99% arsenic removal from a 5 mg/L solution of As­(V) up to a filtering volume of 65 mL. For As­(III), it maintained 95% adsorption efficiency up to a 30 mL volume. The filter exhibited decent adsorption capacity, 4.5 and 3.1 mg/g for As­(III) and As­(V), respectively, in relation to filter weight. However, when the capacity is normalized to the weight of the active filler, the adsorption efficiency of 30.2 and 21.1 mg/g for As­(III) and As­(V) was determined, respectively. This indicates that the developed filter exhibits a higher adsorption capacity for As­(III) than As­(V). The most notable feature of the filter is its ability to retain efficiency in the presence of competitive anions. For both arsenic species, more than 90% of removal was retained. The filter also successfully reduced the concentration of arsenic from 2.5 mg/L to the WHO limit of 10 μg/L in a simulated groundwater system for volumes up to 70 mL, showing its effectiveness in real-world groundwater.

## Supplementary Material



## Data Availability

The data supporting the findings of this study are available throughout the manuscript and in the supporting files.

## Notes

EDX-spectra of PS-CS/MOF(15) in Figure S1, adsorption isotherms of arsenic species in Figure S2, Temkin adsorption isotherm fittings in Figure S3, and Temkin isotherm fitting results in Table S1 (PDF)
