# Supplementary material for: Selective Laser Sintering of a Chitosan/MOF Composite: 3D-Printed Adsorbent for Arsenic
Source: ACS Omega. 2025 Nov 11;10(46):55461–70. doi: 10.1021/acsomega.5c06075 (PMC12658808; doi:10.1021/acsomega.5c06075)
Supplement: Supplementary file 1 [file ao5c06075_si_001.pdf]

Supporting Information

Selective Laser Sintering of a Chitosan/MOF Composite: 3D-Printed Adsorbent for Arsenic

Jessy Joseph[a], Ari Väisänen[a], Manu Lahtinen[a*]

[a] *Department of Chemistry, University of Jyväskylä, P.O. Box 35, FI-40014, Jyväskylä, Finland*

Corresponding author: Manu Lahtinen, manu.k.lahtinen@jyu.fi

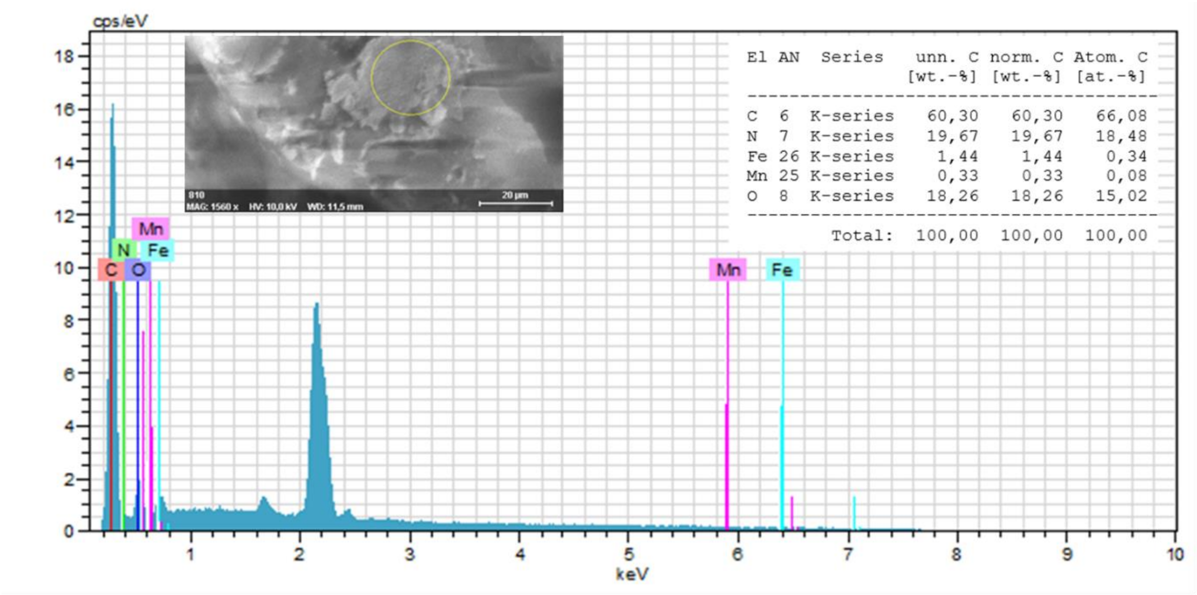

Figure S1. EDX-spectra of PS-CS/MOF(15)

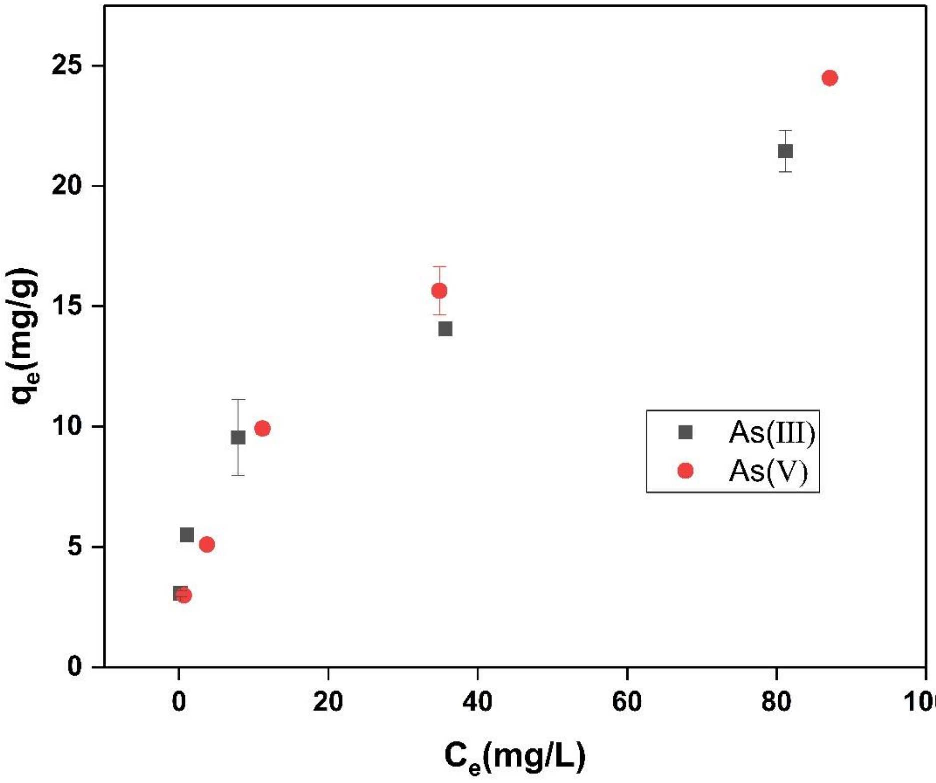

Figure S2 Adsorption isotherm of arsenic species for the developed PS-CS/MOF(15).

The Temkin adsorption isotherm is described by the equation,

$$q_e = B ln(AC_e) \qquad \text{Eq: S1}$$

Where, C_e (mg/L) is the equilibrium concentration of arsenic species, q_e (mg/g) is the equilibrium adsorption capacity. A Temkin binding equilibrium constant (L/g) and B Temkin constant related to the heat of adsorption (J/mol).

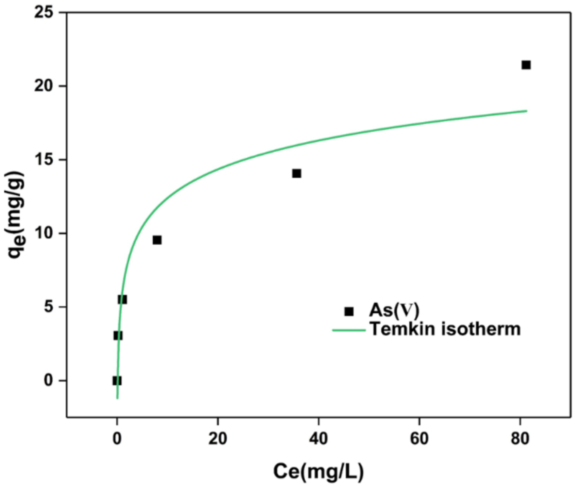

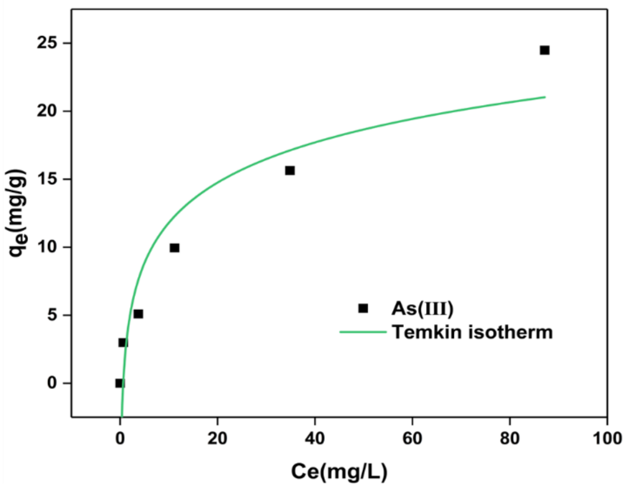

Figure S3. Temkin adsorption isotherm fitting of PS-CS/MOF(15) for As(III) and As(V) adsorption

Table S1. The fitted Temkin adsorption isotherm parameters of the PS-CS/MOF(15) for As(III) and As(V) adsorption

Temkin adsorption isotherm parameter	As(V): relative to filler	As(III): relative to filler
A (L g^{-1})	8.059	1.595
B (J mol^{-1})	2.823	4.260
R^2	0.932	0.9176
